# Burden of epilepsy in rural Kenya measured in disability-adjusted life years

**DOI:** 10.1111/epi.12741

**Published:** 2014-07-31

**Authors:** Fredrick Ibinda, Ryan G Wagner, Melanie Y Bertram, Anthony K Ngugi, Evasius Bauni, Theo Vos, Josemir W Sander, Charles R Newton

**Affiliations:** * KEMRI/Wellcome Trust Research Programme, Centre for Geographic Medicine Research – CoastKilifi, Kenya; † MRC/Wits Rural Public Health and Health Transitions Research Unit (Agincourt), Faculty of Health Sciences, School of Public Health, University of the WitwatersrandAcornhoek, South Africa; ‡ Epidemiology and Global Health, Department of Public Health and Clinical Medicine, Umeå UniversityUmeå, Sweden; § World Health OrganizationGeneva, Switzerland; ¶ Research Support Unit, Faculty of Health Sciences, Aga Khan University (East Africa)Nairobi, Kenya; # Institute of Health Metrics and Evaluation, University of WashingtonSeattle, Washington, U.S.A; ** NIHR University College London Hospitals Biomedical Research Centre, Department of Clinical and Experimental Epilepsy, UCL Institute of NeurologyLondon, United Kingdom; †† Epilepsy SocietyChalfont St Peter, United Kingdom; ‡‡ SEIN–Stichting Epilepsie Instellingen NederlandHeemstede, The Netherlands; §§ Neurosciences Unit, Institute of Child Health, University College LondonLondon, United Kingdom; ¶¶ Department of Psychiatry, University of OxfordOxford, United Kingdom

**Keywords:** Burden, Disability-adjusted life years, Epilepsy, Remission, Treatment gap

## Abstract

**Objectives:**

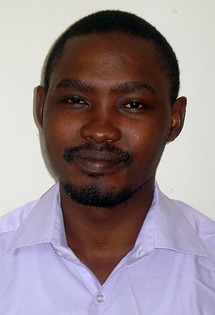
The burden of epilepsy, in terms of both morbidity and mortality, is likely to vary depending on the etiology (primary [genetic/unknown] vs. secondary [structural/metabolic]) and with the use of antiepileptic drugs (AEDs). We estimated the disability-adjusted life years (DALYs) and modeled the remission rates of active convulsive epilepsy (ACE) using epidemiologic data collected over the last decade in rural Kilifi, Kenya.

**Methods:**

We used measures of prevalence, incidence, and mortality to model the remission of epilepsy using disease-modeling software (DisMod II). DALYs were calculated as the sum of Years Lost to Disability (YLD) and Years of Life Lost (YLL) due to premature death using the prevalence approach, with disability weights (DWs) from the 2010 Global Burden of Disease (GBD) study. DALYs were calculated with R statistical software with the associated uncertainty intervals (UIs) computed by bootstrapping.

**Results:**

A total of 1,005 (95% UI 797–1,213) DALYs were lost to ACE, which is 433 (95% UI 393–469) DALYs lost per 100,000 people. Twenty-six percent (113/100,000/year, 95% UI 106–117) of the DALYs were due to YLD and 74% (320/100,000/year, 95% UI 248–416) to YLL. Primary epilepsy accounted for fewer DALYs than secondary epilepsy (98 vs. 334 DALYs per 100,000 people). Those taking AEDs contributed fewer DALYs than those not taking AEDs (167 vs. 266 DALYs per 100,000 people). The proportion of people with ACE in remission per year was estimated at 11.0% in males and 12.0% in females, with highest rates in the 0–5 year age group.

**Significance:**

The DALYs for ACE are high in rural Kenya, but less than the estimates of 2010 GBD study. Three-fourths of DALYs resulted from secondary epilepsy. Use of AEDs was associated with 40% reduction of DALYs. Improving adherence to AEDs may reduce the burden of epilepsy in this area.

Epilepsy is common in low and middle income countries (LMICs),^1^ and is associated with considerable mortality and morbidity^2^,^3^ and poor quality of life.^4^,^5^ The treatment gap (the proportion of people with epilepsy [PWE] who do not receive appropriate treatment) is large (31–100%), especially in LMICs.^6^–^8^

The World Bank introduced the disability-adjusted life year (DALY) metric, which incorporates both the morbidity and mortality of a disease.^9^,^10^ DALYs are the sum of Years Lived with Disability (YLD) and Years of Life Lost (YLL) due to premature death. The 2010 Global Burden of Disease (GBD) study estimated that primary epilepsy (cause of epilepsy either genetic or unknown) accounted for 253 DALYs per 100,000 people globally,^11^ which amounts to 0.75% of the total GBD. The disability weights (DWs) used to calculate the YLD component of the DALYs seek to quantify the health loss from nonfatal diseases and injuries. The DWs used in the 2010 GBD study ranked severe epilepsy^12^ higher than HIV.^13^

A study from rural China reported that epilepsy caused 208 DALYs per year per 100,000 people (67 YLD, 141 YLL).^14^ The Kenyan estimate from the GBD 2010 is 550 (95% uncertainty interval [UI] [380–750]) DALYs per 100,000 people; http://viz.healthmetricsandevaluation.org/gbd-compare/. Because the GBD estimates may not be directly extrapolated to specific regions within a country due to different socioeconomic settings, and only estimated the burden for primary epilepsy, studies are needed to assess the accuracy of GBD estimates locally and to inform resource allocation for the prevention and treatment of both primary and secondary epilepsy.

We established a cohort of people with active convulsive epilepsy (ACE) from two cross-sectional studies conducted over a 5-year interval (2003 and 2008) in Kilifi, Kenya. We studied ACE, since this type of epilepsy is most reliably detected in community-based studies and is associated with the greatest mortality and disability. The unadjusted incidence was 37.6 (95% UI 32.7–43.3) per 100,000 persons per year,^15^ and the crude prevalence of ACE was 3.0 (95% UI 2.8–3.2) per 1,000 people.^16^ The mortality rate was 33.3 (95% UI 25.9–42.8) per 1,000 persons per year with a standardized mortality ratio of 6.5.^17^ We have not previously estimated the percentage of people with ACE who remit or the DALYs due to epilepsy.

We used data from Kilifi, Kenya, to estimate the remission and the burden of ACE in terms of YLD, YLL, and the DALYs. We also computed the DALYs for primary (genetic/unknown) and secondary (structural/metabolic) epilepsy, and for those taking and not taking antiepileptic drugs (AEDs).

## Methods

### Definition of terms

ACE was defined as having at least two unprovoked convulsive seizures, with at least one seizure within the last 12 months, which is the criterion for starting antiepileptic treatment in Kenya.^18^ We classified ACE as either primary (genetic/unknown) or secondary (structural/metabolic) epilepsy based on the proximate cause.^19^ Those with undetermined epilepsy were not included in the analysis. We classified as secondary epilepsy those who had identifiable structural/metabolic causes as suggested by history, for example, head injury or documented infection of the central nervous system, and/or detection of focal abnormalities on clinical and/or electroencephalographic examination, in accordance with the International League Against Epilepsy (ILAE) epidemiology guidelines.^20^ This information was complete for 747 (99.1%) of the people with ACE. We have conducted magnetic resonance imaging (MRI) on 152 of these patients who have focal epilepsy, defined as ictal discharges on electroencephalography and focal clinical features that are suggestive of identifiable underlying causes. We did not have adequate data for classifying seven cases, and these were classified as having undetermined etiology.^20^ We classified the remainder as having primary epilepsy. Primary epilepsy was defined as the absence of identifiable structural/metabolic causes. Epilepsy was classified as being of unknown etiology if adequate evaluation of the available data were not suggestive of a genetic and secondary cause.

### Study setting

This study uses data from Kilifi, a rural area on the Kenyan Coast. The framework of the study was within the Kilifi Health and Demographic Surveillance System (KHDSS), which covers an area of 189 km^2^, with about 260,000 residents. The KHDSS has been used to define the prevalence, incidence, and mortality of epilepsy, as well as other common diseases. A community census is carried out every 4–6 months to obtain vital statistics,^21^ including migrations, births, and deaths.

### Data sources (input parameters)

#### Population structure and mortality

During a population survey in 2008, we identified 754 people with ACE from the 232,164 people in the KHDSS and followed them up for 3 years. Data on sociodemographic and clinical characteristics as well as deaths and causes of death were collected. This information was used to construct age and sex population distribution and the number of deaths in people with and without epilepsy (Table1). We used prevalence, incidence, and case fatality to estimate the remission and duration of ACE using the software program DISMOD II (World Health Organization, Geneva, Switzerland), in which remission is defined as seizure free for >1 year.^22^

**Table 1:** Population, total deaths in the population, total deaths in people with epilepsy, and deaths directly attributable to epilepsy in Kilifi 2008–2010

Age-group (years)	Population composition		Deaths in the population		People with ACE		Deaths in people with ACE		Deaths in people with ACE directly attributable to epilepsy	
	Female	Male	Female	Male	Female	Male	Female	Male	Female	Male
0–5	20,759	21,107	130	171	42	55	3	1	0	0
6–12	26,053	26,194	64	75	76	88	4	5	1	5
13–18	16,691	17,535	42	57	75	83	4	6	2	4
19–28	20,658	16,861	107	74	87	73	9	5	7	3
29–49	25,610	16,660	370	274	53	56	4	6	1	4
50+	14,137	9,899	959	1,020	35	31	8	6	3	4
Total	123,908	108,256	1,672	1,671	368	386	32	29	14	20

The table shows the distribution of 232,164 people in 2008; 754 had active convulsive epilepsy. By 2010, 3,343 people died, 3,282 without epilepsy and 61 with epilepsy. There were 34 deaths that were found to be directly attributable to epilepsy.

#### Prevalence

We used a 2008 cross-sectional survey to determine the prevalence of ACE. The study employed a three-stage screening process to identify people with ACE.^23^ Prevalence was calculated as the ratio of clinically confirmed cases to the population screened. Similarly, prevalence of primary and secondary epilepsy was calculated as the ratio of the presumed cases with primary and secondary epilepsy to the total population screened.

#### Incidence

An original three-stage cross-sectional survey from 2003 identified people with and without ACE, who were followed for 5 years. The incidence rate used in this study is based on incident cases identified during the 2008 cross-sectional study.^16^,^24^ Incidence was calculated as the number of incident cases divided by the person years of observation (PYO) among the individuals identified during both studies. There were no data for the 0–5 year band in the 2003 survey, as it focused on older children and adults. Consequently the incidence rate for this age band could not be calculated. Because the two studies were 5 years apart, we assumed that those aged 5 years and younger with prevalent epilepsy in the 2008 survey were all incident cases over the 5-year period, and to calculate the incidence rate the number of cases was divided by PYO (in this case PYO was calculated as 2.5 multiplied by the number of children younger than 5 years of age in the 2008 screened population, which is based on the assumption that the date of births were normally distributed over the 5 years). Details on cohort identification are published elsewhere.^15^

### Case fatality

This is based on a cohort of 232,164 people; 754 diagnosed as having ACE were followed from December 2007 with a median (interquartile range) follow-up period of 32.6 (27.7–41.1) months, whereas the general population was followed for 32.6 (28.5–32.6) months. During this period there were 3,343 deaths in the total cohort; 61 of these were people with ACE. The putative causes of death were determined using World Health Organization (WHO) verbal autopsy tool, which was administered through a trained fieldworker to a carer/guardian of the diseased.^17^ Three trained clinicians independently assigned the cause of death, and whenever there were disagreements, another coder (Charles Newton, neurologist) was involved as an independent reviewer. The fourth reviewer examined the records, and any discrepancies were resolved through consensus; if consensus was not reached, the cause of death was unclassified. Cause of death was defined as epilepsy-related if death occurred during a prolonged seizure or seizure-related accident or was sudden without a preceding illness.^17^ While computing the case fatality for input into DisMod II, deaths that could not be directly attributed to epilepsy (excess deaths) were not considered. Case fatality was computed as the number of deaths (directly attributable to epilepsy) divided by the number of people with ACE. Similarly, only the deaths that were attributable to epilepsy were used while computing the YLL, but these were divided by the period of follow-up in years.

### Analysis

#### Remission of epilepsy using DisMod II

**Figure 1:**
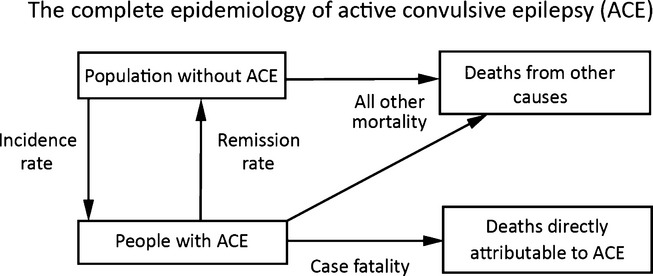
This diagram represents the complete epidemiology of active convulsive epilepsy (ACE). It shows the interrelationship among the different states through the hazards (incidence, remission, and mortality).

Data on sex- and age-specific population distribution, population mortality, incidence, prevalence, and case fatality were entered into the public domain disease-modeling software (DisMod II) from WHO (http://www.who.int/healthinfo/global_burden_disease/tools_software/en/). DisMod II checks the internal consistency of the input parameters and uses a set of differential equations to model the complete epidemiology of a disease using the inter-relationships between the input variables as shown in Figure1.^22^ Data were smoothed using inbuilt DisMod mathematical functions, specifically piecewise linear interpolation and moving average. In constructing the uncertainty intervals, 1,000 iterations of the DisMod model were run each time, selecting values from the uncertainty distributions around the input parameters. Results are presented in six age bands (0–5, 6–12, 13–18, 19–28, 29–49, and 50+ years) for consistency with prior studies published in the same region.^15^,^16^ The proportion of PWE who remitted per year was calculated as 1 − e^(−remission rate)^, since DisMod II outputs remission rates.

#### Disability-adjusted life years

YLL, YLD, and DALYs and their bootstrapped uncertainty intervals (percentile; with 1,000 iterations) were calculated in R, an open-source software for statistical computing and graphics.^25^ Bootstrapping was implemented using the R boot package.^26^,^27^ DALYs were computed using the prevalence approach consistent with the GBD 2010 studies. YLLs for each age group were computed as the product of the deaths directly attributable to epilepsy and the standard life expectancy for that age category. The standard life expectancies were computed from the new standard reference life table that has a life expectancy at birth of 86 years for both males and females and was used in GBD 2010 studies.^28^ YLD for each age group were calculated as the product of the prevalence and the disability weight (DW). Calculation of YLL, YLD, and DALYs for primary and secondary epilepsy was based on their respective prevalence ratio and deaths that were directly related to them, with further analysis examining the effect of taking AEDs.

The calculation of YLD employs the use of DWs. From the recent GBD studies, there were four DWs associated with epilepsy: 0.072 for treated seizure-free epilepsy, 0.319 for treated epilepsy with recent seizures, 0.420 for untreated epilepsy, and 0.657 for severe forms of epilepsy.^12^ We used the 2010 GBD study's mean DW for epilepsy (0.346) for the sub-Saharan African region (T. Vos, personal communication). Because the three-stage methodology used to screen for people with ACE had relatively low sensitivity (46.8%), we also provide DALY estimates based on adjusted prevalence. Unless explicitly stated, the term DALYs refers to those computed with unadjusted prevalence.

## Results

The modeled prevalence of ACE was 3.2 per 1,000 people (95% UI 1.1–5.1) for males and 2.6 (95% UI 0.9–4.4) for females. Incidence estimates were 48.0 (95% UI 10.9–51.8) and 39.2 (95% UI 7.5–41.9) per 100,000 people per year in males and females, respectively. The standardized mortality ratios (SMRs) were significantly higher in males (10.8, 95% UI 9.3–11.9) than females (8.1, 95% UI 8.1–8.5), with the highest values in those aged 6–12 years for males and 50+ for females (Table2). The proportion of people in whom epilepsy remitted per year was estimated at 11.0% (95% UI 0.5–22.2%) in males and 12.0% (95% UI 0.8–22.5%) in females. A higher proportion of people with ACE who remitted were younger, particularly in those aged 0–5 years at 29.8% per year in males and 30.2% per year in females (Table2).

**Table 2:** Prevalence, incidence, mean duration of disease, remission, and standardized mortality ratio estimated in DisMod II for epilepsy in Kilifi, 2008

Age (years)	Prevalence per 1,000	Incidence per 100,000/year	Standardized mortality ratio	Instantaneous remission rate (%)	Proportion remitting per year
Male					
0–5	2.31 (0.82–3.81)	85.19 (29.05–93.32)	3.23 (3.08–3.25)	35.39 (3.02–68.82)	29.81 (2.97–49.75)
6–12	2.90 (1.03–4.84)	53.07 (7.42–56.75)	16.95 (16.40–17.94)	11.19 (0.00–30.50)	10.59 (0.00–26.29)
13–18	3.84 (1.24–6.62)	39.64 (6.26–42.72)	15.07 (13.39–16.05)	5.80 (0.00–30.50)	5.64 (0.00–26.29)
19–28	3.97 (1.11–5.92)	28.53 (5.93–30.39)	8.87 (8.84–9.34)	6.59 (0.00–13.54)	6.38 (0.00–12.66)
29–49	3.12 (0.99–4.97)	24.07 (6.30–25.64)	5.87 (5.43–6.17)	5.68 (0.00–11.59)	5.52 (0.00–10.94)
50+	3.13 (1.25–5.16)	43.52 (6.05–46.73)	8.72 (8.21–9.27)	8.59 (0.00–17.62)	8.23 (0.00–16.15)
All ages	3.16 (1.05–5.14)	48.00 (10.92–51.80)	10.80 (9.30–11.90)	11.60 (0.46–25.13)	10.95 (0.46–22.22)
Female					
0–5	1.86 (0.63–3.10)	69.44 (14.50–74.37)	1.70 (1.60–1.70)	35.93 (5.59–66.84)	30.18 (5.44–48.75)
6–12	2.42 (0.97–3.92)	48.20 (7.60–50.88)	6.88 (6.67–7.23)	12.74 (0.66–28.61)	11.96 (0.66–24.88)
13–18	3.71 (1.29–6.14)	41.36 (6.77–44.35)	9.79 (9.37–10.26)	5.36 (0.00–10.96)	5.22 (0.00–10.38)
19–28	3.58 (1.20–6.00)	33.83 (5.56–36.69)	7.75 (6.48–7.93)	11.71 (0.00–23.84)	11.05 (0.00–21.21)
29–49	2.06 (0.66–4.10)	14.95 (4.74–15.96)	2.97 (2.79–3.04)	8.13 (0.00–15.94)	7.81 (0.00–14.73)
50+	2.12 (0.89–3.42)	27.30 (5.46–29.31)	9.91 (9.53–10.47)	9.59 (0.01–19.51)	9.14 (0.01–17.72)
All ages	2.59 (0.92–4.41)	39.16 (7.47–41.89)	8.13 (8.06–8.53)	12.82 (0.79–25.47)	12.03 (0.79–22.49)

To obtain the proportion remitting per year the following formula was used 1 − e^(−remission rate)^.

The burden of ACE is provided in both DALYs and DALY rates (Tables3 and S1). The total number of DALYs associated with ACE were estimated at 1,005 (95% UI 797–1,213), which is 433 (95% UI 393–469) DALYs per 100,000 people. One hundred and thirteen (95% UI 106–117) DALYs per 1,000 people, or 26% of overall DALYs, were attributed to YLD, whereas 320 (74%; 95% UI 248–416), were attributed to YLL. As shown in Table4, the burden of epilepsy was significantly higher in males (535 DALYs per 100,000 people) than females (344 DALYs per 100,000 people). The highest burden in terms of DALYs was observed in people aged between 19 and 28 years at 799 per 100,000 people (95% UI 388–997) (Table3).

**Table 3:** Active convulsive epilepsy YLL, YLD, and DALYs per 100,000 population (95% uncertainty intervals) by age and sex in Kilifi 2008

Age	YLL	YLD	DALYs
Male			
0–5	0 (0–0)	89 (85–128)	89 (75–93)
6–12	553 (337–1,015)	118 (107–141)	670 (549–1,341)
13–18	592 (295–893)	164 (148–174)	756 (340–1,041)
19–28	434 (135–434)	150 (135–185)	584 (276–636)
29–49	480 (119–918)	116 (100–127)	596 (356–695)
50+	440 (331–668)	108 (98–133)	548 (309–567)
All ages	411 (390–443)	123 (113–129)	535 (397–684)
Female			
0–5	0 (0–0)	68 (51.7–90)	68 (55–73)
6–12	108 (108–215)	100 (96–112)	207 (89–308)
13–18	314 (0–626)	156 (122–189)	472 (133–503)
19–28	828 (477–1,183)	147 (136–152)	975 (734–1,823)
29–49	76 (0–152)	72 (68–78)	147 (81–156)
50+	196 (0–310)	86 (69–91)	282 (171–448)
All ages	241 (141–333)	103 (97–112)	344 (287–387)
Male and female			
0–5	0 (0–0)	79 (74–89)	79 (80–92)
6–12	331 (220–554)	109 (104–120)	439 (324–718)
13–18	456 (226–458)	161 (141–167)	617 (387–1,004)
19–28	651 (199–894)	149 (129–158)	799 (388–997)
29–49	235 (187–389)	89 (79–91)	324 (129–388)
50+	297 (249–342)	95 (78–104)	392 (309–687)
All ages	320 (248–416)	113 (106–117)	433 (393–469)

**Table 4:** Primary and secondary epilepsy YLL, YLD, and DALYs per 100,000 population (95% uncertainty intervals) by age and sex in Kilifi 2008

Age (years)	Primary epilepsy			Secondary epilepsy		
	YLL	YLD	DALYs	YLL	YLD	DALYs
0–5	0 (0–0)	28 (24–35)	28 (22–37)	0 (0–0)	49 (41–65)	49 (47–54)
6–12	54 (0–54)	22 (20–28)	76 (19–130)	277 (220–497)	85 (89–95)	363 (188–523)
13–18	75 (0–150)	25 (20–30)	100 (16–327)	381 (154–611)	133 (120–155)	515 (360–667)
19–28	258 (316–464)	29 (20–32)	287 (88–614)	393 (327–591)	119 (95–145)	512 (374–580)
29–49	46 (0–92)	21 (20–25)	66 (16–161)	189 (47–257)	69 (57–85)	258 (214–353)
50+	0 (0–0)	25 (9–27)	25 (23–33)	297 (120–449)	71 (62–86)	367 (185–800)
All ages	73 (40–131)	25 (23–26)	98 (57–137)	247 (222–315)	87 (85–90)	334 (303–376)

One fifth of people with ACE (165/754, 21.9%) had primary epilepsy, 582 (77.2%) had secondary epilepsy, and 7 (0.9%) had causes that were not diagnosable. The prevalence of primary epilepsy was 0.7 per 1,000 (95% UI 0.6–0.8), whereas that of secondary epilepsy was 2.5 per 1,000 people (95% UI 2.3–2.7). Primary epilepsy contributed lower DALYs (98 [95% UI 57–137] DALYs per 100,000 people) than secondary epilepsy (334 [95% UI 303–376] DALYs per 100,000 people) (Table4). Forty-four percent (329/754) of those with ACE reported taking AEDs and were found to contribute significantly lower DALYs (167 [95% UI 136–198] DALYs per 100,000 people) than those who were not taking AEDs (266 [95% UI 288–333] DALYs per 100,000 people) (Table5).

**Table 5:** Active convulsive epilepsy YLL, YLD, and DALYs per 100,000 population (95% uncertainty intervals) by age and sex, and whether on antiepileptic drugs in Kilifi 2008

Age	Not taking antiepileptic drugs epilepsy			Taking antiepileptic drugs epilepsy		
	YLL	YLD	DALYs	YLL	YLD	DALYs
0–5	0 (0–0)	54.5 (48–61)	55 (50–63)	0 (0–0)	24 (22–26)	24 (20–30)
6–12	223 (54–390)	66.9 (58–76)	290 (123–354)	107 (0–107)	42 (36–50)	149 (95–149)
13–18	303 (229–305)	78 (70–91)	381 (223–605)	153 (0–307)	83 (83–104)	236 (63–397)
19–28	326 (201–459)	76 (65–86)	401 (198–532)	325 (130–519)	73 (66–86)	398 (131–462)
29–49	184 (47–231)	48 (40–52)	231 (87–598)	51 (0–103)	42 (37–46)	93 (39–102)
50+	205 (114–197)	59 (39–81)	264 (175–311)	91 (46–139)	36 (30–46)	127 (120–181)
All ages	202 (138–331)	63 (59–67)	266 (288–333)	118 (83–173)	49 (50–53)	167 (136–198)

After adjusting the prevalence estimates for the sensitivity of the three-stage methodology, the total number of DALYs associated with ACE were estimated at 1,301 (95% UI 1,154–1,435), which is 561 (95% UI 522–740) DALYs per 100,000 people. Primary epilepsy contributed 126 (95% UI 82–143) DALYs and secondary epilepsy 433 (95% UI 386–490) DALYs per 100,000 people. People not taking AEDs contributed 338 (95% UI 311–420) DALYs, whereas those taking AEDs contributed 223 (95% UI 205–272) DALYs per 100,000 people.

## Discussion

This study provides modeled estimates of the remission rate and the burden of primary and secondary epilepsy and examines the potential influence of AED treatment on the burden in rural Kenya. We calculated DALYs using a prevalence-based approach by inputting available epidemiologic data collected over a decade in this area. The highest burden occurred in some of the most economically productive ages (13–28 years). Remission was estimated at 11% per year in males and 12% in females, with the highest rates in the 0–5 years old at 30% per year. The computed DALYs for ACE were 433/100,000 people, whereas those for secondary epilepsy were three times more than for primary epilepsy. PWE not taking AEDs contributed 62% of DALYs, compared to 38% from those taking AEDs.

The adjusted DALYs associated with ACE in this study are high. This could be ascribed to the use of data from a resource-poor country, which has high prevalence, incidence, treatment gap, and mortality due to epilepsy. In a recent meta-analysis, we showed that the median lifetime prevalence of ACE in rural areas is three times that reported in urban areas of developing countries.^29^ This high prevalence could be due to high endemicity of associated parasitic risk factors and perinatal problems.^1^ With the exception of the 19–28 age group, our DALYs estimates were slightly higher in males than females, a result of the significantly higher mortality observed in males than females (SMR of 10.8 in males [95% UI 9.3–11.9] compared to 8.1 [95% UI 8.1–8.5] in females).

Secondary epilepsy contributes more to the overall burden of epilepsy than primary epilepsy, which, again, is a reflection of higher mortality in those with secondary epilepsy (27 deaths) than in those with primary epilepsy (7 deaths) and also greater severity of illness (higher DWs). It is likely that acquired epilepsy has a much higher mortality due to the severity of the underlying causes, such as tumors, stroke, and encephalopathies. The comparison of DALYs from primary epilepsy (adjusted and unadjusted) in this study, differ from the GBD estimate for Kenya of 550 (95% UI 380–750) DALYs per 100,000 people (http://viz.healthmetricsandevaluation.org/gbd-compare/). This difference could suggest that the country GBD estimates cannot be generalized to specific rural parts of a country. In addition, it could be ascribed to the fact that the GBD study estimated the burden of both convulsive and nonconvulsive epilepsy (with nonconvulsive epilepsies forming 44% of all epilepsies), although it did not include some secondary causes, for example, stroke, infections of the central nervous system, or perinatal problems.^11^,^20^

The YLL, YLD, and DALYs computed by Ding et al.^14^ from rural China could not be compared with our estimates, because they calculated DALYs using the incidence approach. The prevalence approach has been favored over the incidence approach by the GBD 2010 team because of greater data availability and ability to adjust for comorbidity. The two methods of DALY computation yield different results.^30^ It is, however, likely that the burden is higher in rural Kenya than in China because of the larger treatment gap in Kilifi,^6^ compared to the mixed locations (urban and rural areas) studied in China.^8^

Among PWE not taking AEDs, mortality was a major contributor to the DALYs lost, with 22 deaths compared to 12 deaths in those who were on AEDs. A comparison of the DALYs for those taking and not taking AEDs suggests that the burden of epilepsy may be substantially reduced by reducing the high treatment gap in LMICs.^6^,^7^ This confirms the findings by Chisholm et al.^31^ that up to 40% of the burden of epilepsy could be reduced by increasing the AED treatment coverage. In particular, phenobarbital and phenytoin—AEDs widely available in LMICs—are the most cost-effective based on efficacy and cost of acquisition.^31^ It is likely that even more DALYs occur in people who are not taking AEDs in our study, as information on AED usage was based on self-reporting, which has been shown to be unreliable because it often overestimates adherence to AEDs.^6^

The modeled remission for both sexes are low compared to those proposed in a review on the natural history of epilepsy.^32^ In this cohort, 425/754 (56.4%) (0–5 years 8.8%; 6–12 years 13.4%; 13–18 years 10.2%; 19–28 years 10.9; 29–49 years 7.7%, and 50+ years 5.4%) were not taking AEDs, suggesting that, in most individuals, remission had occurred spontaneously. In an earlier study, we found that failure to seek medical treatment and nonadherence to AEDs was associated with the cost of AEDs, patients’ traditional religious beliefs, negative attitudes about medical treatment, living far from health facilities, and negative attitudes about epilepsy.^6^ The high remission rate in the 0–5 year age group, compared to the other age bands, is similar to what was observed in Ecuador,^33^ and supports the finding that early onset epilepsy tends to remit early in the course of the condition. The low remission rate may also be associated with higher proportions of severe epilepsy in Kilifi, which is suggested by the substantial proportion of PWE with learning difficulties (27.5%), neurologic deficits (17.6%), focal neurologic deficits (16.7%), and long duration of epilepsy (40.9%), all of which have been shown to be associated with intractable epilepsy.^34^,^35^

The major strength of this study is in the use of data from previous epilepsy studies carried out in the same area and with consistent definitions of epilepsy, which is likely to yield reliable and representative estimates of the burden of epilepsy in rural Kenya.^3^ There are some limitations. First, we only examined ACE, which is likely to underestimate the burden of epilepsy, as it accounts for only about a third to half of all epilepsies.^36^ Second, we had to estimate the incidence in the under-fives using the 2008 cross-sectional study, which may differ from actual values. Finally, the sensitivity of the verbal autopsy tool was not measured, but a physician-certified verbal autopsy has been shown to have a high sensitivity and specificity for sickle cell disease as well as the five major causes of death in Kilifi.^37^,^38^

## Conclusion

The DALYs for ACE are high, particularly in the 6 to 28-year-old age group. The GBD 2010 overestimates the burden of primary epilepsy in rural Kenya. Secondary epilepsy was associated with three times more DALYs than primary epilepsy, and being on AEDs reduced the DALYs by about 40%. Improving adherence to AEDs, thereby reducing the treatment gap, would likely reduce the burden of epilepsy in this area.

## Supporting Information

Table S1 YLL, YLD, and DALYs lost due to active convulsive epilepsy by age group and sex with the associated 95% uncertainty intervals (UIs).
