# Supplementary material for: Burden of epilepsy in rural Kenya measured in disability-adjusted life years
Source: Epilepsia. 2014 Jul 31;55(10):1626–33. doi: 10.1111/epi.12741 (PMC4238788; doi:10.1111/epi.12741)
Supplement: Table S1 — YLL, YLD, and DALYs lost due to active convulsive epilepsy by age group and sex with the associated 95% uncertainty intervals (UIs). [file epi0055-1626-SD1.docx]

Supplementary table 1: YLL, YLD and DALYs lost due to active convulsive epilepsy by age group and sex with the associated 95% uncertainty intervals.

| Males | | | |
| --- | --- | --- | --- |
| Age | YLL | YLD | DALYs |
| 0-5 | 0(0-0) | 18.7 (13.1 - 20.4) | 18.7 (16.3 - 22.5) |
| 6-12 | 144.7 (0 - 174.4) | 30.8 (23.2 - 31.5) | 175.5 (58.8 - 350.7) |
| 13-18 | 103.8 (51.4 - 259) | 28.7 (25.9 - 30.8) | 132.5 (54.1 - 136.6) |
| 19-28 | 73.1 (48.1 - 189.1) | 25.3 (21.8 - 28.7) | 98.4 (23.2 - 173.1) |
| 29-49 | 80.0 (21.7 - 140.2) | 19.4 (19.4 - 22.8) | 99.3 (59.5 - 164.6) |
| 50+ | 43.5 (25.6 - 62.8) | 10.7 (8.3 - 14.5) | 54.3 (10.0 - 54.6) |
| All ages | 445.2 (316.3 - 488.1) | 133.6 (131.8 - 135.6) | 578.7 (357.4 - 606.4) |
| Females | | | |
| Age | YLL | YLD | DALYs |
| 0-5 | 0(0-0) | 14.2 (8.3 - 15.2) | 14.2 (12.1 - 15.2) |
| 6-12 | 28.0 (0 - 56.0) | 25.9 (24.2 - 27.3) | 54 (22.1 - 136.0) |
| 13-18 | 52.4 (26.0 - 130.5) | 26.3 (24.6 - 30.4) | 78.7 (53.0 - 103.0) |
| 19-28 | 171.0 (97.8 - 268.8) | 30.4 (25.6 - 32.9) | 201.4 (124.5 - 253.9) |
| 29-49 | 19.4 (0 - 77.6) | 18.3 (16.3 - 20.8) | 37.7 (17.0 - 75.9) |
| 50+ | 27.8 (5.8 - 50.0) | 12.1 (10.0 - 14.5) | 39.9 (17.6 - 61.8) |
| All ages | 298.6 (205.9 - 385.4) | 127.3 (119.7 - 125.6) | 425.9 (376.8 - 466.6) |
| Males and females | | | |
| Age | YLL | YLD | DALYs |
| 0-5 | 0(0-0) | 32.9 (29.4 - 32.9) | 32.9 (26.3 - 32.9) |
| 6-12 | 172.8 (86.7 - 283.9) | 56.7 (51.9 - 62.6) | 229.5 (113.7 - 269.3) |
| 13-18 | 156.2 (51.4 - 286.4) | 55 (52.9 - 63) | 211.2 (99.5 - 236.9) |
| 19-28 | 244.1 (169.7 - 364.7) | 55.7 (50.9 - 64.7) | 299.8 (201.7 - 472.3) |
| 29-49 | 99.4 (39.5 - 182.3) | 37.7 (33.9 - 39.4) | 137.1 (99.3 - 176.0) |
| 50+ | 71.3 (31.1 - 85.7) | 22.8 (20.1 - 26.6) | 94.1 (82.1 - 106.2) |
| All ages | 743.7 (640.3 - 883.6) | 260.9 (242.2 - 272.3) | 1004.6 (797.0 - 1212.6) |
